# Temperature Changes during Implant Osteotomy Preparations in Fresh Human Cadaver Tibiae, Comparing Straight with Tapered Drills †

**DOI:** 10.3390/ma15072369

**Published:** 2022-03-23

**Authors:** Nikolaos Soldatos, Laura Nelson-Rabe, Nathan Palanker, Nikola Angelov, Georgios Romanos, Robin Weltman

**Affiliations:** 1 Department of Periodontics and Dental Hygiene, School of Dentistry, University of Texas, Health Science Center at Houston, 7500 Cambridge Str, Suite 6400, Houston, TX 77054, USA; lauranelsonrabe@gmail.com (L.N.-R.); nathan.palanker@gmail.com (N.P.); nikola.angelov@uth.tmc.edu (N.A.); rlwtx1111@gmail.com (R.W.); 2 Department of Periodontology, LASER Education at SDM, School of Dental Medicine (SDM), Stony Brook University, South Dr, Stony Brook, Long Island, NY 11794, USA; georgios.romanos@stonybrookmedicine.edu; 3 Department of Clinical Sciences, School of Dental Medicine, University of Nevada, Las Vegas, NV 89106, USA

**Keywords:** temperature change, implant drill, osseointegration, straight, tapered, cadaver tibia

## Abstract

The success of osseointegration depends on many factors. With temperatures beyond a 47 °C threshold over 1 min, bone survival may be impaired. The purpose of the study was to evaluate, in fresh human cadaver tibiae, the temperature changes during osteotomy preparations using two straight and two tapered implant systems’ drills, external irrigation, and varying revolutions per minute (RPM). The tibiae from a fresh female cadaver were harvested bilaterally. Two tapered and two straight design drills were assessed. Two-hundred and forty osteotomies were prepared at 6 mm depth following the drill sequence of the manufacturers’ protocol for each drilling speed. Difference in temperature (ΔΤ) was calculated by subtracting the baseline from the maximum temperature (ΔT = T_max_ − T_base_). Drill design and drill diameter, as independent variables or synergistically, had a significant effect on ΔΤ. *Tapered drills:* As the drill diameter increased, ΔΤ increased at all RPM. *Straight drills:* As the drill diameter increased, ΔΤ remained constant or slightly decreased at all RPM. Drill diameter and design had a significant effect on ΔΤ in human tibiae, which never exceeded the critical threshold of 47 °C. Tapered drills caused significantly greater heat production compared to straight drills.

## 1. Introduction

The literature is replete with publications on delineating factors which affect the osseointegration process during preparation of the implant osteotomy and after implant placement [1,2,3,4,5,6,7,8]. The cutting action of the implant drills, while preparing the osteotomy, converts mechanical work into frictional heat energy [9,10,11]. Due to the low thermal conductivity of bone, the energy accumulates at the osteotomy site, raising the local temperature [12,13]. Temperatures beyond a threshold of 47 °C for one minute have been shown to cause protein denaturation, enzyme inactivation, osteocyte necrosis, bone resorption, and delays in regeneration [8,14,15,16,17,18]. Excessive heat may lead to hyperemia, fibrosis, necrosis, osteolytic degeneration, and increased osteoclastic and osteoblastic activity in osseous tissues [19,20,21,22,23]. 

Irrigation (internal, external, or combined) is recognized as the single most effective way to reduce the temperatures during implant osteotomy preparation [21,24,25,26]. However, the use of a surgical guide, the drill diameter, the drill design, the drill material, the wearing and duration of drilling, and the presence and thickness of the cortical bone may all increase the temperature of the alveolar bone [10,18,22,27,28,29,30,31]. Based on the existing literature, authors disagree as to whether high or low revolutions per minute (RPM) positively or negatively alter temperature [22,30,31,32,33,34,35,36]. Additionally, implant manufacturers develop proprietary drill designs with varying surgical protocols. Clinicians would benefit from a comparison of temperature changes with differing drill designs and speeds to decide which manufacturer to utilize in their clinical practices.

Synthetic bone blocks or animal models attempt to simulate the biomechanical responses to human bone injury [14,15,19,21,29,31,32,33,34,35,36,37,38,39,40,41]. Differences in bone macro- and microstructure, composition, and remodeling may differ when comparing animal models with human bone [42]. While an in-vivo human model is preferred for measuring temperature changes during osteotomy preparation, there are limitations with regards to the sterilization of instruments (especially the thermocouple), the quantity of available bone, and the bone density between patients. Therefore, an innovative and highly translational model using fresh human cadaver tibiae immediately postmortem, developed from one of the authors (NS), was used in the present study. 

The purpose of the present study was to evaluate, in fresh human cadaver tibiae, the temperature changes (ΔΤ) during osteotomy preparations using four different implant systems’ drills (two straight, two tapered), with external irrigation and varying RPM. The null hypothesis was that osteotomy preparations at 800 RPM, with external irrigation, will produce the same heat as 1000 and 1200 RPM, in two different implant drill macro-designs (straight or tapered). The specific aims of the study were: (1) to compare ΔΤ in fresh human cadaver tibiae with osteotomy preparations at drilling speeds of 800, 1000 and 1200 RPM; and (2) to compare ΔΤ for two straight and two tapered drill designs as the osteotomy width was gradually increased following the various manufacturers’ drilling protocols. 

## 2. Materials and Methods

No Institutional Review Board (IRB) approval was required for the completion of the present ex vivo fresh human cadaver study. The cadaver was donated for clinical and research purposes to the Department of Neurobiology and Anatomy, McGovern Medical School, University of Texas, Health Science Center at Houston. The relatives signed all the appropriate informed consents, and the cadavers were examined through blood testing to ensure the safety of the present study. 

**Figure 1:**
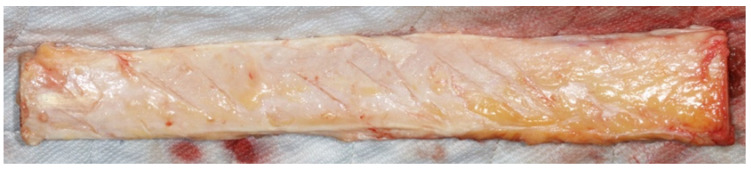
A six-inch-long tibial section, before the placement into a 37 °C water bath, to simulate normal body temperature.

The methodology was reviewed and approved by an independent statistician. The study was funded by the Department of Periodontics and Dental Hygiene, School of Dentistry, University of Texas Health Science Center at Houston, and the implant drills were donated by three dental implant companies. A female deceased patient, aged seventy-four (74), was obtained four hours post-mortem. The time of death was 9:37 am and the patient arrived at the UT Morgue at 1:27 p.m. The patient was deceased due to complications from pneumonia and had previously obtained a do-not-resuscitate code in case of cardiopulmonary arrest. The patient had no history of osteoporosis, diabetes mellitus, bone disease, HIV, hepatitis, or cancer. The fresh, unembalmed tibiae were harvested bilaterally on the day of arrival at 1:35 pm. The overlying skin and muscle tissues were removed from the tibiae exposing the periosteum of the tibial bone. Four sections of six-inches length were prepared and placed into a 37 °C water bath, to simulate normal body temperature (Figure 1).

Calibration: Calibration of the operators (LR, NS) was completed at the beginning of the study, on a type II bone block analog (Saw bones^®^, Vashon island, WA, USA) maintained in the range of 95.2 °F to 99.6 °F (35.1–37.5 °C). All operators took turns preparing osteotomies using a pilot drill separate from the study. Temperature was recorded before and after each osteotomy. This process was repeated until 8 consecutive ΔT measurements fell within 2 °C of one another and the operators’ average ΔT was within 1 °C. 

**Figure 2:**
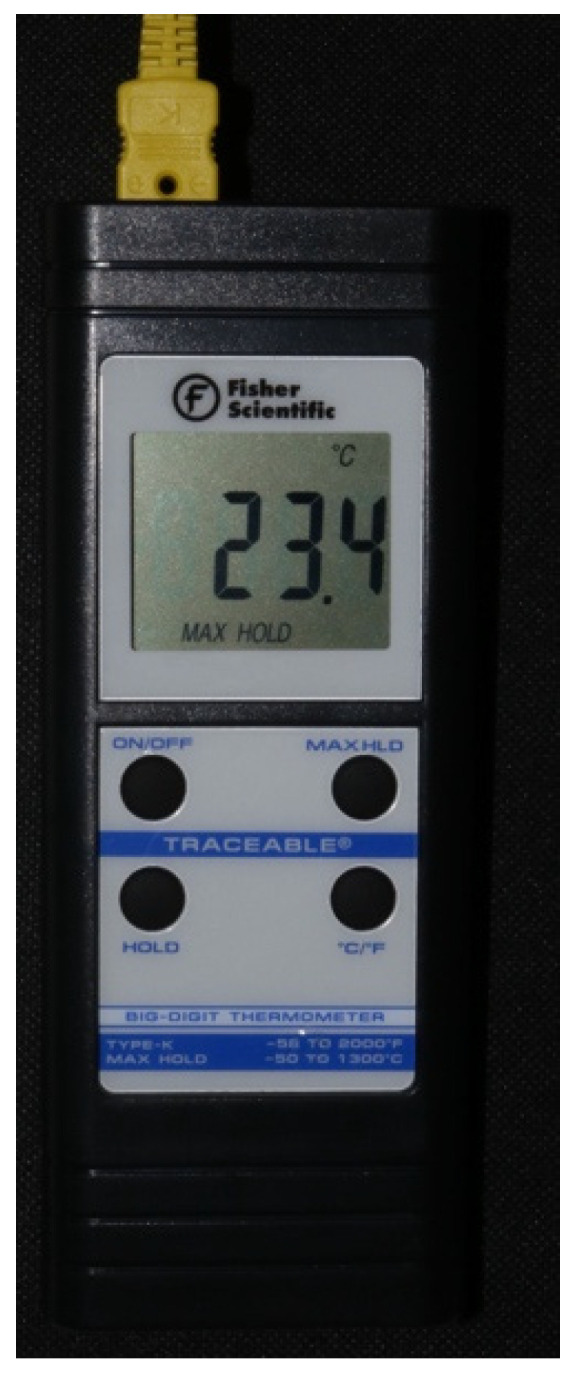
K-type thermocouple (Fisher Scientific 15-078-187, range −58 to 2000 °F, resolution 0.1°/1°, sampling rate 2.5 times per second) with an ultra-fast response naked bead probe (maximum range 260 °C) which was used to measure the temperature.

**Figure 3:**
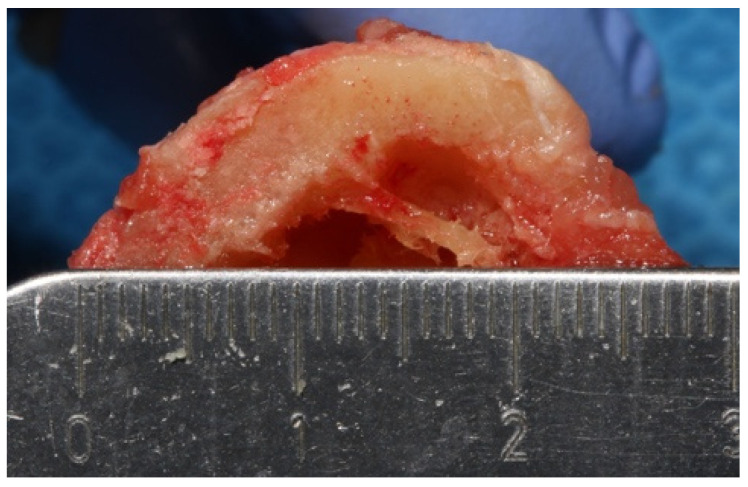
Six (6) mm cortical thickness of the tibiae.

Room temperature was kept at a constant 71 °F (21.6 °C). A Whip-Mix temperature-regulated water bath, filled with phosphate buffered saline (PBS), was maintained at 98.6 °F (37 °C). The temperature of the saline bath was recorded with a Medline (Northfield IL) standard oral thermometer (REF MDS9950) and a K-type thermocouple (Fisher Scientific, Hampton, NH, USA 15-078-187, range −58 to 2000 °F, resolution 0.10/10, sampling rate 2.5 times per second) with an ultra-fast response naked bead probe (maximum range 260 °C) (Figure 2). The cortical thickness was measured and determined to be 6 mm wide (Figure 3). This measurement provided the final depth of drilling without perforating the cortical plate. 

**Figure 4:**
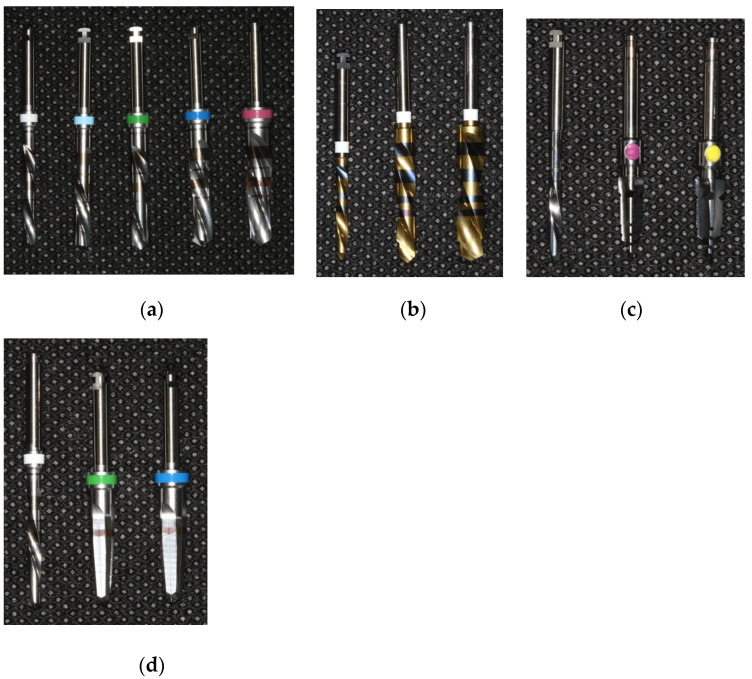
The two straight (**a**,**b**) and the two tapered (**c**,**d**) implant drills used in the study.

Four groups were included in the study: two straight (*†) and two tapered (‡≠) implant drills [* Astra Tech, Dentsply^®^, York, PA, USA † Premium, Sweden & Martina^®^, Due Carrare, Italy ‡ Nobel Biocare^®^, Klote, Switzerland ≠ Shelta, Sweden & Martina^®^, Due Carrare, Italy (Figure 4a–d)]. 

The osteotomies were prepared following the manufacturers’ protocol of drill order, for the placement of a dental implant with a diameter of 5 mm × 6 mm (Table 1). 

**Figure 5:**
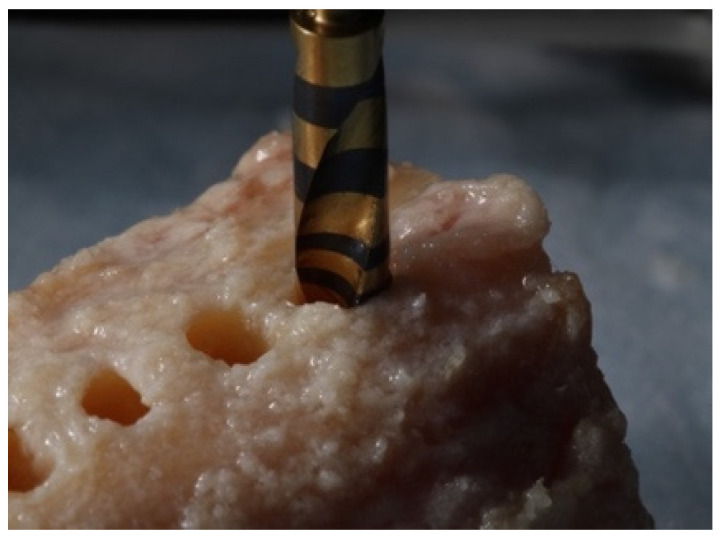
Each osteotomy was prepared with at least 2 mm of bone separating adjacent channels and at 2 mm from the external tibial edge to prevent the dissipation of heat from surrounding osteotomies.

Two-hundred and forty osteotomies (240) were prepared: 20 osteotomies per drill, at each RPM (800, 1000, and 1200 RPM) with external irrigation of sterile 0.9% sodium chloride saline. Each osteotomy was prepared with at least 2 mm of bone separating adjacent channels and at 2 mm from the external tibial edge to prevent dissipation of heat from surrounding osteotomies (Figure 5). 

In addition, osteotomies were sequentially drilled on opposite ends of the bone to allow the surrounding tissues to cool back to normal body temperature prior to successive drilling. 

**Figure 6:**
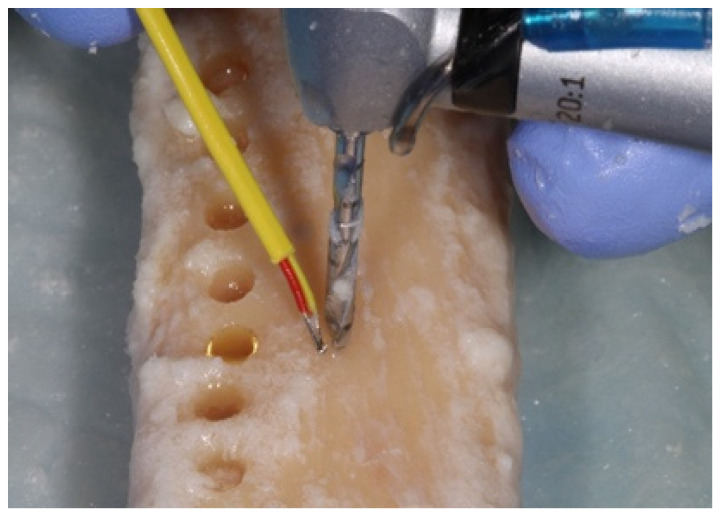
The initial temperature of the bone was recorded with the use of the K-Type thermocouple holding the thermocouple probe against the bony outer cortical layer (**Step 1**).

The initial temperature of the bone was recorded with a K-Type thermocouple (Fisher Scientific 15-078-187, range −58 to 2000 °F, resolution 0.10/10, sampling rate 2.5 times per second) by holding the thermocouple probe against the bony outer cortical layer (**Step 1**) (Figure 6). 

**Figure 7:**
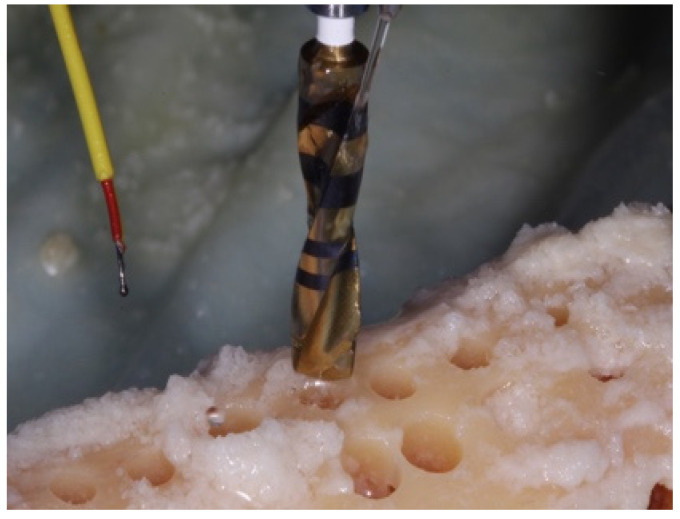
Each osteotomy was completed starting with the pilot drill and with external irrigation at each of the 800, 1000, or 1200 RPM drilling speeds (**Step 2**).

Each osteotomy was completed starting with the pilot drill with external irrigation at each of the 800, 1000, or 1200 RPM drilling speeds (**Step 2**), (Figure 7). 

**Figure 8:**
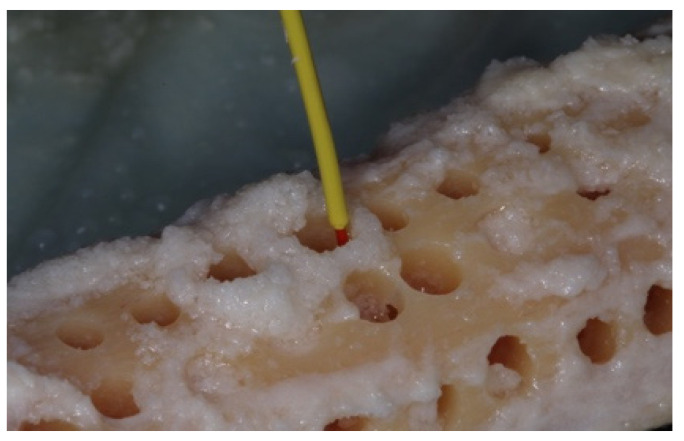
Immediately after osteotomy preparation to the 6 mm depth, the thermocouple probe was inserted along the osteotomy wall and floor and the highest temperature value was recorded (**Step 3**).

Studies reported differences in the bone temperature (1.5 °C) when the thermocouple was placed 0.5 mm lateral from the osteotomy site [12,17]. The finding was in accordance with the findings of the present study; therefore, any attempts to measure the temperature away from the osteotomy site were not applied. For each drill diameter in the drilling sequence, immediately after osteotomy preparation to the 6 mm depth, the thermocouple probe was inserted along the osteotomy wall and floor and the highest temperature value was recorded (**Step 3**) (Figure 8). 

The bone was allowed to cool back to its original starting temperature prior to subsequent drilling (range 10–30 s). The drilling protocol was continued to enlarge the osteotomy to size for a 5 mm diameter implant. During each successive drilling, the initial and maximum bone temperatures were registered at a depth of 6 mm. The existing literature supports the use of drills up to 50 times without surface corrosion or degradation [41,43,44]. However, in our study, the implant drills were only used up to 20 times and then discarded. All values were compiled in an Excel^®^ spreadsheet for further statistical analyses. Variations in temperature were calculated by subtracting the baseline temperature from the maximum temperature (ΔT= T_max_ − T_baseline_). 

Data analyses: All statistical analyses were performed using R statistical software (R 3.02), Vienna, Austria (R Core Team 2017) [45]. A generalized linear model (GLM) analysis was performed, specifying a gamma distribution, to evaluate the effect in ΔΤ using three variables: (i) the drill design, (ii) the drill diameter, and (iii) RPM. Explanatory variables were examined individually for their interactive effect on ΔΤ and synergistically in one-, two-, and three-way interactions. 

## 3. Results

**Figure 9:**
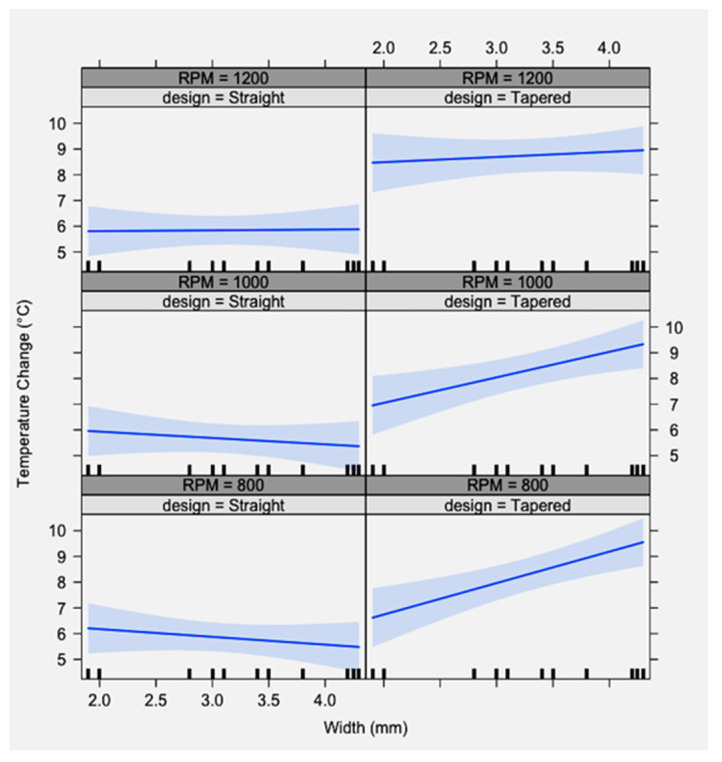
The *Y*-axis shows the temperature change (ΔΤ), and the *X*-axis shows the drill width. As drill diameter increased in a tapered design drill, ΔΤ increased. As the drill diameter increased in a straight design drill, ΔΤ remained constant or slightly decreased.

Figure 9 depicts the results of the study; the *Y*-axis shows the ΔΤ and the *X*-axis shows the drill width. 

As the drill diameter increased in a tapered design drill, ΔΤ increased. As the drill diameter increased in a straight design drill, ΔΤ remained constant or slightly decreased. Both patterns were consistent between the groups of 800, 1000, and 1200 RPM. Tapered drills caused significantly greater heat generation compared to straight drills during osteotomy preparations. The 95% confidence interval (indicated in light blue) showed that, in both designs and at all RPM, the ΔΤ did not exceed the critical threshold of 47 °C. 

The drill design (tapered or straight) and drill width, as independent variables, were found to significantly affect the temperature. When the variables were tested synergistically, the combination of drill design and drill width significantly affected the temperature. RPM, as a single variable, did not affect the temperature significantly, either independently or synergistically with the drill width and the drill design (Table 2). 

## 4. Discussion

The purpose of the present study was to measure ΔΤ using fresh human cadaver tibiae to simulate the effects of the implant osteotomy preparation in human bone. Human tibiae and mandibular bone, although having different origins, possess similar compressive strength and elastic modulus [46]. Therefore, a fresh human cadaver tibiae model was used in this study. 

This study adds to the breadth of published literature by comparing multiple variables together in a human bone model, which simulates clinical practice. Additionally, the human bone specimens were comprised solely of cortical bone. Previous publications have shown that drilling dense cortical bone generates greater frictional heat with increased local temperatures as compared to cortical bone from various animal models or cortico-cancellous bone, thus providing insight into drilling of type I-II bone [11,27,29,40,42]. The anatomy of edentulous sites has been measured in cadaver models and from CBCT scans [47,48,49,50]. Katranji et al. measured the buccal and lingual cortical plates’ thickness in cadavers along with the intermediary cancellous bone. The cortical plates in the edentulous mandibular sites ranged from 1.5 mm to greater than 2 mm and slightly narrower plates in the edentulous maxilla. The intermediary cancellous bone in the anterior regions of both mandible and maxilla and the maxillary premolar region measured approximately 2 mm [50]. Relating to clinical applications in implantology, after the initial 2 mm pilot drill in most implant systems, the expansion of the implant osteotomy requires drilling solely in cortical bone [50]. Other studies measured the density and thickness at the cortical and cancellous bone in atrophic edentulous ridges. The incisal/occlusal thicknesses averaged 1–2 mm but included ranges greater than 5 mm, with the apical cortical plate averaging 7 mm in thickness [47,48,49,50]. The cancellous bone density and bone volume fractions were most significant in the anterior mandible [47,48]. Mense et al. found that 20% of the patients evaluated had no cancellous bone between the buccal and lingual cortical plates, indicating that all drilling would be in cortical bone [49]. 

Analysis of the variables individually found that both drill design (tapered versus straight) and drill diameter significantly affected ΔΤ. The performance of multiple variable statistical analyses found the combination of design and width significantly affected temperature change. While the straight drills showed relatively constant temperature values during drilling, the tapered design drills showed a trend towards increasing temperature as the drill width increased. The ΔΤ did not exceed 10 °C, which would signify a change from our initial water bath temperature of 37 °C to the necrosis threshold of 47 °C. The methodology utilized in the present study attempted to minimize heat generation due to factors other than drill diameter, rotational speed, and design. All osteotomies were performed by three previously calibrated operators under external irrigation, providing copious amounts of cooled saline to the drill surfaces. New twist drills were used at the start of the experiment and were discarded after 20 osteotomies. Heat generation and drill wear have been shown to be minimal with fewer than 25 drill uses [41,43,44]. The osteotomies were placed solely in cortical bone, utilizing the drilling sequences specified by the tested implant manufacturers, including rotational speeds within the ranges per manufacturer guidelines. 

Temperature changes in bone, due to drilling, have been investigated for decades in the medical and dental literature [8,15,21,22,24]. Comparisons between studies are challenging due to differences in methodologies. For instance, the instruments, which measure bone temperature after osteotomy preparation, vary in the published literature. Both thermocouple and infrared thermal imaging devices have been utilized and positioned at varying distances from, or within, the osteotomies after drilling [9,21,27,50]. Temperature changes after osteotomy preparation have been studied in animal and human models, within varying bone densities [8,27,42]. Thus, when comparing study results within the literature, trends in temperature change, rather than exact values, may be most relevant.

Additionally, beyond differences in the study models, modern statistical algorithms provide the means to evaluate the effects of multiple independent variables on a single dependent variable. The challenge, once again, in relating the results of the present investigation to published literature with multiple variable analyses is finding similar models, as well as variables within the models, to compare.

Scarano et al. used infrared thermography to assess temperature changes in bovine femoral bone immersed in a controlled 26 °C saline bath at 800 RPM with external irrigation [51]. The drill diameters were 3.7 mm with differing drill designs and straight versus tapered (with a tri-flute design). The single drill created the osteotomy, after which bone temperature was measured. While the absolute temperature difference between the two drill designs amounted to 2 °C, statistical analysis found this difference to be significant. The authors suggested that the geometry and number of flutes in the tapered drill influenced bone temperature [47]. While infrared thermography is highly accurate, it is generally not used to measure temperatures through liquids and can be confounded by the external irrigation required for osteotomy preparation. 

Similar to the Scarano et al. study [51], Cordioli and Majzoub [29] compared temperature changes using straight design twist drills or tri-flute shaped drills in bovine cortical bone at a constant drill speed of 1500 RPM with external irrigation. Sequential drilling was performed for each drill design starting with a 2 mm twist drill (straight design) or a 3.3 mm tri-flute designed drill [29]. Thermocouple measurements found that the tri-flute shaped drills, when compared to twist drills, led to significantly lower temperature recordings after osteotomy preparation. Tri-flute burs have cutting edges directed parallel to the long axis of the drill while the cutting edges of the twist burs are perpendicular to the long axis of the drill. The highest heat values were recorded in the 2.0 mm twist drill group when compared to 3.0 mm, 3.3 mm, and 4.0 mm tri-flute drills. The authors proposed that tri-flute drill design provides a better cutting efficiency by virtue of its cutting action along the full length of the flute, leading to greater heat dissipation [29]. Interestingly, the tapered drills utilized in the present study appear to have cutting surfaces along the length of the drill; however, the resultant temperature change opposes the temperatures reported in the Scarano and Cordioli studies.

To relate thermal alterations to bone healing, Gehrke et al. compared the thermal effects of preparing osteotomies in rabbit tibia with either cylinder or tapered drills [52]. Intermittent or continuous movement of the drills was considered as well, while utilizing the rotational speeds recommended by the manufacturers of the implant systems. Processing of the histologic specimens occurred 30 days after preparation of the osteotomies. In contrast to the present study, Gehrke et al. found that the conical drill sequence produced a significantly smaller increase in temperature during both techniques (continuous and intermittent) and formed more new bone [52]. 

Sharawy et al. evaluated differences in temperature change using four implant systems (two internally irrigated, two externally irrigated) at either 1225, 1667, or 2500 RPM in porcine jawbone. Four K-type thermocouples were placed 1 mm from the periphery of the osteotomy [34]. The highest drilling speed (2500 RPM) consistently produced the smallest amount of temperature changes (ΔT = 4 °C), while the slower speeds produced similar results. The RPM utilized in the present study were within the ranges suggested by the manufacturers, and closer to the 1225 RPM. 

Soldatos et al. utilized a similar methodology to that of the present study. They evaluated three different straight implant drill designs to create osteotomies in a polyurethane foam block of synthetic bone (Saw Bones^®^). Drills were used with and without external irrigation at 800, 1000, and 1200 RPM. The synthetic block was placed into a water bath providing a block temperature of 37 °C prior to drilling. With the use of external irrigation, only the 2 mm diameter pilot drills showed increased temperature over the 47 °C threshold. The straight drills produced a similar trend in temperature change as found in the present study; as the drill diameter increased, the maximum temperature tended to decrease [32]. 

Expanding rotational speeds beyond those of the present study, a recent investigation translated rotational speed variations, drill dimensions, and temperature changes to bone viability [53]. Osteotomies were prepared in rabbit femur using conical shaped drills (3-, 3.5-, and 4-mm diameters) at rotational speeds of 1000, 1500, 2000 RPM, or a combination of 2000 RPM for the first two drills (3- and 3.5-mm diameter) and 1000 RPM for the final 4 mm diameter drill with external irrigation. The manufacturer of the implant system recommended a rotational speed of 1200 RPM for all diameter drills. Histologic specimens were prepared immediately after the surgical procedures to test for the disappearance of viable osteocytes adjacent to the cutting surfaces. Less heat was produced at the higher rotational speeds as well as with the wider diameter drills. Similarly, osteocytes with nuclei were found in greater numbers adjacent to the cut surfaces at sites prepared with the wider diameter drills, and at the higher speeds. The authors suggested that utilizing slower speeds require more drilling time creating more frictional heat, in turn increasing the potential for osteocyte death. 

In the present study, the null hypothesis was rejected since during osteotomy preparations with external irrigation, tapered drills caused significantly greater heat generation, compared to straight drills at 800, 1000, and 1200 RPM. There are some limitations regarding the present study. While an in vitro simulation of osteotomy preparation provided an acceptable model for this study, an in vivo comparison would produce a more accurate representation of a clinical setting. Sterilization of the thermocouple and standardization of the patients were the major challenges to proceeding with an in vivo study. The sterilization process disrupted the various parts and confounded precise temperature readings. In addition, the implant osteotomy preparations in the present study were on cortical bone only. An in vivo study would take into consideration the cancellous bone and the blood flow. The blood flow could influence heat dissipation, even though the literature, interestingly, does not support this statement [10]. A future recommendation should include histological, histomorphometrical, and immunohistochemical analyses of the specimens to better understand the healing response in a cellular level following each drilling protocol.

In summary, comparisons of the present study to the body of published literature show varying trends. All reports agree that drill design, drill dimensions, and rotational speed influence heat production. Though not statistically significant in the present study, for cylinder/straight drills, temperatures tended to decrease as the drill diameter increased. The tapered (or conical) drills in the present study showed the opposite trend—that temperature increased with increasing diameter. However, the temperature elevations did not reach the bone necrosis threshold of 47 °C. Other studies have shown differing trends with tapered drills [51,52,53]. The rotational speeds tested herein were within the range recommended by the manufacturers of the implant systems. Other studies presented in this discussion tested speeds much higher (up to 2500 RPM). In theory, the faster speeds provide a shorter contact time with bone yielding less frictional heat. Clinicians must consider all these variables when preparing osteotomies. The cause of implant failures may be multifactorial. Minimizing local heat generation translates to improved bone metabolism, which is critical for implant success.

## 5. Conclusions

The conclusions of the study:

- The drill diameter and design had a significant effect on changes in bone temperature in a fresh human cadaver tibial model, which never exceeded the critical threshold of 47 °C.
- The drill speed did not play a significant role in altering temperature.
- The tapered drills caused significantly greater heat production compared to straight drills.

## Figures and Tables

**Table 1:** The osteotomies were prepared following the manufacturers’ protocol of drill order for a 5 mm diameter dental implant.

Implant Designs	Drilling Protocol				
ASTRA Tech^®^ straight (York, PA, USA)	1.9 mm	2.5/3.1 mm	3.7/4.3 mm		
Nobel Biocare^®^ tapered (Klote, Switzerland)	2.0 mm	3.5 mm	4.3 mm		
Sweden & Martina Implantology^®^ straight (Premium) (Due Carrare, Italy)	2.0 mm	2.8 mm	3.0 mm	3.4 mm	4.25 mm
Sweden & Martina Implantology^®^ tapered (Shelta) (Due Carrare, Italy)	2.0 mm	3.8 mm	4.2 mm		

**Table 2:** Analysis of deviance; one-, two-, and three-way interactions between the variables.

Source	SS	df	F	*p* Value
**Design**	28.4	1	98.9	<0.001 *
RPM	0.2	2	0.4	<0.68
**Width**	1.2	1	4.3	<0.04 *
Design/RPM	0.1	2	0.2	<0.79
**Design/width**	2.2	1	7.5	<0.01 *
RPM/width	0.3	2	0.6	<0.55
Design/RPM/width	0.9	2	1.7	<0.18

* Indicates statistical significance.

## Data Availability

Data are available upon request from the corresponding author.
